# 

**Published:** 1865-01

**Authors:** 


### MEDICAL SOCIETY OF THE STATE OF NEW YORK.

The Fifty-Eighth Annual Meeting of the Medical Society of the State of New York will be held in Albany, on Tuesday, Wednesday and Thursday, the 7th, 8th and 9th days of February, 1865. A full attendance is requested. Credentials of new delegates should state from what date to what date the delegate is appointed.

SYLVESTER D. WILLARD, M. D.,
Secretary.

### BOOKS RECEIVED.

*A Treatise on Military Surgery and Hygiene.* By FRANK HASTINGS HAMILTON, M. D., *late Lieutenant-Colonel, Medical Inspector U. S. A.; Professor of Military Surgery and Hygiene, and of Fractures and Dislocations, in Bellevue Medical College; Surgeon to Bellevue Hospital; Professor of Military Surgery, &c., in Long Island College Hospital; author of "Treatise on Fractures and Dislocations," and of a "Practical Treatise on Military Surgery," illustrated with* 127 *engravings. New York:* BAILLIERE BROTHERS, 520 *Broadway,* 1865.

